# Interactions of atrazine with transition metal ions in aqueous media: experimental and computational approach

**DOI:** 10.1007/s13205-015-0281-x

**Published:** 2015-02-06

**Authors:** Vijay Kumar, Virender Kumar, Niraj Upadhyay, Sitansh Sharma

**Affiliations:** 1 Department of Chemistry, Lovely Professional University, Phagwara, 144002 Punjab India; 2 Department of Chemistry, Dr. Hari Singh Gour University, Sagar, Madhya Pradesh India

**Keywords:** Atrazine, Metal complex, FTIR analysis, Water of hydration, Chelation

## Abstract

Transition metal ions have their own significances and utility. Externally applied pesticides may alter the bioavailability of these metal ions to plants through the coordinating ability of these pesticides with metal ions. In current study a series of metal complexes containing atrazine (Atr) group(s) attached to metal(II) (M) frame, with the formula; [M(Atr)_n_.xH_2_O.yCl] (where M = Mn, Fe, Co, Ni, Cu or Zn; *n* = 1 or 2; *x* = 1–4; *y* = 1–2), have been synthesized for the first time to check the interactions of atrazine with transition metal ions. More importantly, all the complexes were synthesized at neutral pH in aqueous medium. The major differences among the FTIR spectra were observed between 3,700–2,800 and 1,800–1,350 cm^−1^. On the basis of FTIR, CHN and computational study, it was observed that Mn, Ni and Cu formed complexes in 1:2 and Fe, Co and Zn in 1:1. The obtained results were supported by 3D molecular modeling using GAMESS computations as a package of ChemBio3D Ultra14 program. The FTIR spectral analysis and 3D molecular modeling suggests that the Atr can show coordination through the nitrogen (in between two side chains) of ring as well as nitrogen (non steric amine) of side chain with different metal ions.

## Introduction

2-chloro-*N* ^*4*^-*N* ^*6*^-isopropyl-1,3,5-trizine-4,6-diamine or atrazine (Atr) is a broad spectrum herbicide (EPA 2005; Meng and Carper 2000). It inhibits photosynthesis and interferes with other enzymatic processes of weeds. It is the member of triazine family and it is still used in about 90 countries all over the world (EPA 2005; Meng and Carper 2000). Annual use of atrazine was estimated to be 80,000 tons worldwide. Since, it is the well-known fact that transport of pesticides in environment occurs by their electrical potentials, adsorption and complex-formation powers (Kumar et al. 2013a, b; Kumar et al. 2014; Meng and Carper 2000; Prasad et al. 2013), same may be applicable for atrazine too.

Atr previously has been the theme of theoretical metal complex studies (Meng and Carper 2000). Theoretical studies show that atrazine can form complexes with different metal ions that are one metal ion to one Atr molecule (1:2) and one metal ion to two Atr molecules (1:2). Such metal to Atr complexes may include water of hydration coordinated to the metal ions in complexes. The formation of dimers suggests that, there is a chance of metal complex formation. As per our best information, no experimental study on transition metal complex of Atr is reported in literature. Because of diverse applications of transition metal ions like Mn, Fe, Co, Ni, Cu, Zn for the healthy growth of plants and humans, therefore, it is found interesting to investigate the complexation of Atr with series of divalent transition metal ions.

## Experimental

### Material and instruments

All the chemicals used were analytical reagent grade. The complexes were prepared as procedure described below. The C, H, N analysis for all the samples were carried out. The FTIR spectra of KBr discs were recorded on a Shimadzu-8400 s FTIR spectrophotometer. To collect good resolution and fine spectra of synthesized metal complexes, each and every time 1 mg of each product was added to 100 mg of KBr. To weigh the above mentioned quantity of metal complex and KBr, weighing balance with five digits was used.

All the FTIR spectra including spectra of Atr were compared to each other using ATR spectral analysis. For the comparative ATR analysis of metal-Atr complexes with Atr, all the FTIR spectra were set to baseline and converted to ATR spectra using FTIR software tool of Shimadzu-8400 s FTIR spectrophotometer.

### Procedure to synthesize atrazine–metal ions complexes

Aqueous solution (10 ml) of metal salts (1 mM) was added to ethanolic solution (10 ml) of atrazine (2 mM), the pH of reaction mixture was adjusted at ~7 using NaOH solution. The resulting solution was stirred for 3 h on a magnetic stirrer at 70 °C, followed by concentrating it to one-third of its volume. Fine amorphous products were collected, washed with hot water (to remove unreacted metal ions) followed by ethanol (to remove unreacted atrazine or also act as drying agent), dried in vacuum desiccator (to remove excess water content) and allowed for FTIR and elemental analysis. After performing the solubility test of the synthesized complexes it was observed that complexes were partially soluble in chloroform and DMSO only. The melting points of all the complexes were between 190 and 260 °C.

### Molecular modeling studies

An attempt to gain a better insight on the molecular structure of Metal complexes, geometric optimization and conformational analysis has been performed by the use of Merck Molecular Force Field 94 (MMFF94) program (Esperdy and Shillady 2007; Hakobyan et al. 2014). All the calculations refer to isolated molecules in vacuum. To calculate the above mentioned parameters, executable program file of GAMESS (Cambridge Software ChemBio3D Ultra 14.0.) program was run on PC (Esperdy and Shillady 2007; Hakobyan et al. 2014).

## Result and discussion

**Fig. 1:**
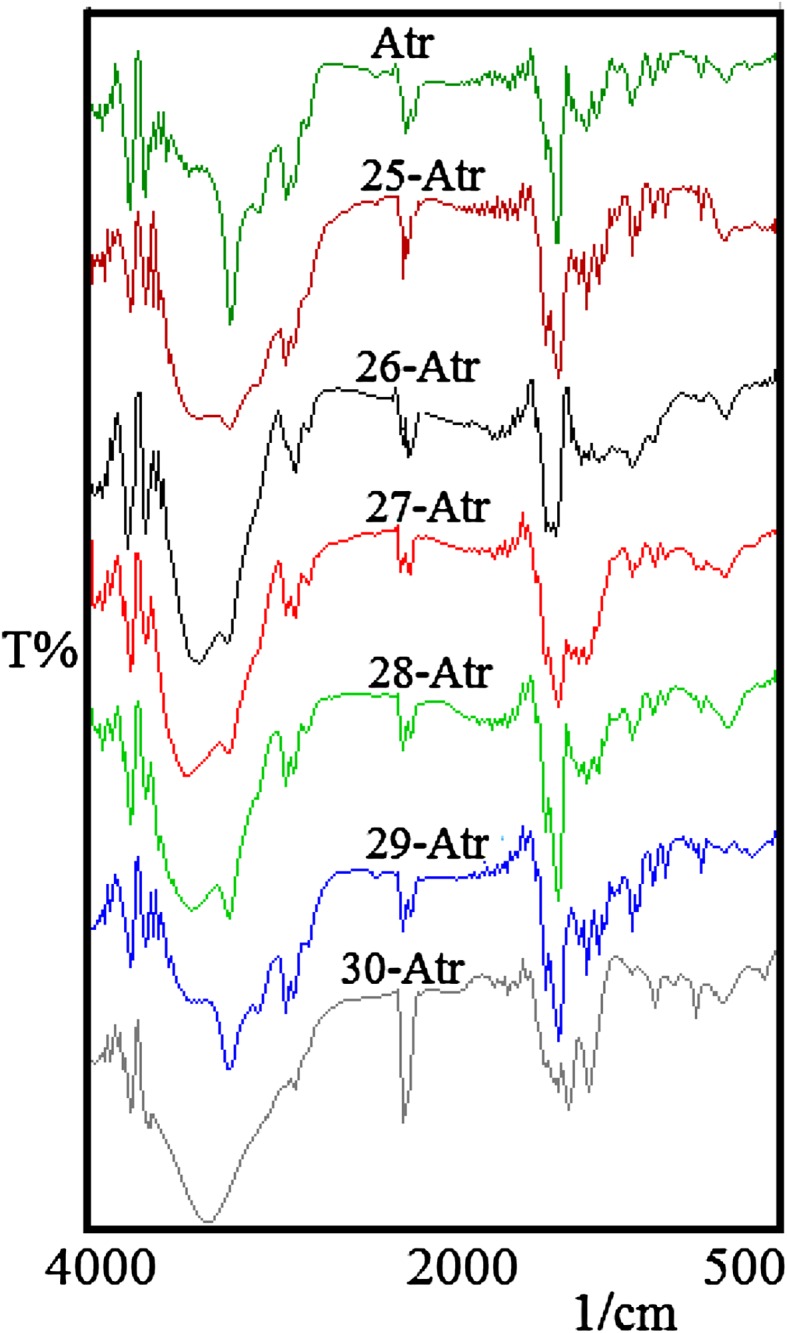
FTIR spectra of atrazine metal complexes

The comparative FTIR spectra of Atr and its metal complexes have shown the shifting in wavenumber and decrease of intensity in the stretching band of the ν(NH), and ν(CN) at 3,320–3,210 & 1,650–1,585, and 2,190–2,050 & 1,110–1,010 cm^−1^ (Fig. 1; Table 1). The IR spectra of complexes were showing absorption bands centered at 683–412 cm^−1^, indicating the presence of M–N and M–O bonds (metal water bond) (Fig. 1; Table 1). Also, strong absorptions bands observed for all the complexes at 750–840 cm^−1^ were due to the “out of plane” (oop) vibrations of the NH of Atr. Moreover, a strong absorption band at 710–560 cm^−1^ for complexes was due to an O–M–N stretching vibration, indicating an O–M–N bonding or water-M-Atr bonding (Nakamoto 1986).

**Table 1:** Change in vibrational frequencies of Atrazine after metal complexation

Assignment	Atr	Mn-Atr	Fe-Atr	Co-Atr	Ni-Atr	Cu-Atr	Zn-Atr
*v*(O–H)	–	3,422^m^	3,414^sh^	3,481^m^	–	3,460^m^	3,439^b^
*v*(N–H)	3,261^vs^	3,265^vs^	3,269^vs^	3,265^vs^	3,265^m^	3,261^s^	–,
	3,115^w^	3,120^w^	3,110^sh^	3,120^sh^	3,119^w^	3,115^sh^	–,
*v*(C–H)	2,974^m^	2,972^s^	2,968^m^	2,972^m^	2,974^m^	2,974^m^	2,983^m^
	2,929^m^	2,931^m^	2,926^m^	2,928^m^	2,931^w^	2,929^m^	2,926^w^
	2,854^w^	2,862^m^	2,860^m^	2,858^w^	2,862^w^	2,852^vw^	2,860^w^
δ(N–H) v_1_	1,666^m^	1,660^vs^	1,666^sh^	1,666^sh^	1,660^sh^	1,668^sh^	1,664^sh^
δ(OH) v_2_	1,620^vs^	1,616^vs^	1,614^vs^	1,616 ^m^	1,616^m^	1,622 ^m^	1,627^sh^
*v*(C = N) v_3_	1,575^vs^	1,575^vs^	1,591^vs^	1,568^vs^	1,575^vs^	1,562^sh^	1,588^sh^
	1,558^vs^	1,558^s^	1,572^vs^	–	1,558^vs^	1,548^vs^	1,558^m^
δ(C–H)	1,481^s^	1,475^m^	1,475^sh^	1,477^s^	1,475^sh^	–	1,491^m^
	1,440^s^	1,450^m^	1,425^sh^	1,437^s^	1,450^m^	1,442^m^	–
	1,404^s^	1,400^m^	1,363^sh^	1,398^m^	1,400^m^	1,404^m^	1,398^w^
	1,346^s^	1,342^s^	–	1,342^sh^	1,342^m^	1,346^w^	–
	1,303^m^	1,131^m^	1,307^sh^	–	1,315^sh^	1,305^w^	–
*v*(C-NH)	1,166^s^	1,165^m^	1,151^b^	1,166^w^	1,168^m^	1,169^w^	1,168^w^
	1,134^w^	1,132^w^	–	1,139^sh^	1,134^w^	1,132^sh^	–
*v*(C–N)	1,055^s^	1,053^s^	1,049^s^	1,049^m^	1,053^w^	1,053^m^	1,045^m^
Ring breath	991^s^	991^w^	989^sh^	989^w^	991^w^	991^m^	949^w^
oop(N–H)	804^m^	804^w^	798^sh^	800^w^	802^w^	804^w^	831^w^
γ(C–H)	723^m^	–	–	710^w^	–	–	734^w^
*v*(C–Cl)	677^m^	684^b^	667^b^	677^b^	657^b^	678^sh^	680^b^
δ(ring) v_4_	533^m^	–	549^sh^	547^w^	549^w^	–	520^w^
M-CN	–	2,182^m^	2,185^m^	2,183^w^	–	–	–
	–	2,095^m^	2,095^m^	2,098^w^	–	–	–
	–	2,041^m^	2,045^w^	2,044^m^	–	–	–
M–N	–	535	486	522	493	592	474
M–O	–	432	451	439	418	442	414

*s* strong, *vs* very strong, *b* broad, *vb* very broad, *sh* shoulder, *m* medium, *w* weak

**Fig. 2:**
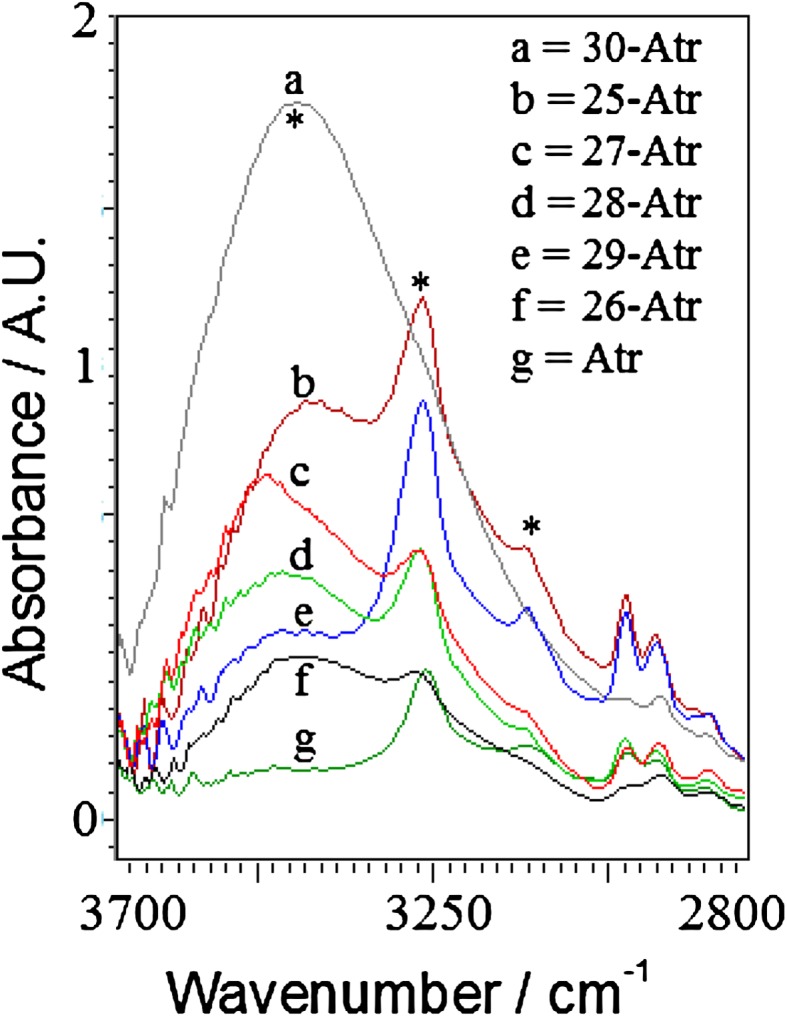
Qualitative change in absorption of Atrazine–metal complexes between 3,700 and 2,800 cm^−1^

**Fig. 3:**
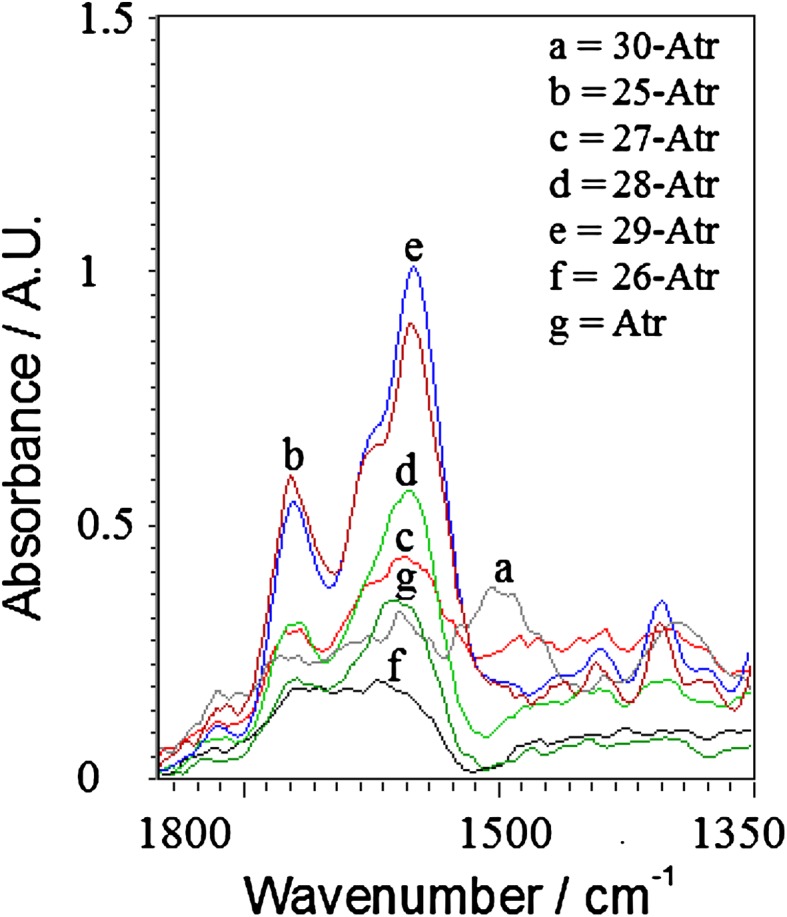
Quantitative change in absorption of Atrazine–metal complexes between 1,800 and 1,350 cm^−1^

On the basis of FTIR spectral features (Table 1; Fig. 1), it was observed that all the FTIR spectra were different over two ranges, these ranges were 3,700–2,800 cm^−1^ and 1,800–1,350 cm^−1^ (Figs. 2, 3). Atrazine has five nitrogen atoms each having a lone pair of electrons to donate. First of all it is necessary to determine whether the coordination occurs through the ring nitrogen or side chain(s) nitrogen or both of them are involved in complex formation. The ring nitrogen (between two substituents at 4th and 6th position of ring) is known to be more basic in comparison to the amino nitrogen. It is a known fact that when the amino or side chain(s) nitrogen atom is involved in complex formation, drastic changes occur in amino group vibrational wave numbers, namely, NH stretching and bending modes shift to lower wave numbers, whereas NH twisting and wagging modes shift to higher wave numbers. On the other hand when the ring nitrogen is involved in complex formation certain vibrational modes increase in value due to both coupling with M–N bond vibrations and alterations of the force field (Medlycott et al. 2008). Especially the main changes have taken place in the stretching frequencies of C=N.

### IR-ATR analysis over a range of 3,700–2,800 cm^−1^

On the basis of Fig. 2 and Table 2, there were three peaks observed at 3,454, 3,266 and 3,124 cm^−1^ due to stretching frequencies of OH and NH. The OH stretching frequencies at 3,454 cm^−1^ was due to the presence of water molecules. The NH stretching frequencies at 3,266 and 3,124 cm^−1^ were due to the presence of secondary amines of Atr molecules. ATR analysis at 3,454 cm^−1^ has shown that Zn-Atr has highest absorption or water molecules and Fe-Atr has lowest absorption or water molecules. The order of absorption of metal-Atr complexes at 3,454 cm^−1^ is; Zn > Mn > Co > Cu > Ni > Fe (Fig. 2). IR frequencies in metal complexes at 3,787 and 3,705 cm^−1^ were due to the water of crystal and at 3,454 cm^−1^ was due to the water of coordination. ATR analysis at 3,266 and 3,124 cm^−1^ has shown that Mn-Atr has highest absorption or minimum involvement of NH of Atr and Fe-Atr has lowest absorption or highest involvement of NH of Atr. The order of absorption of metal-Atr complexes at 3,266 and 3,124 cm^−1^ is; Mn > Zn > Cu > Co ~Ni ≫ Fe (Fig. 2).

**Table 2:** Quantitative change in vibrational frequencies of atrazine after metal complexation w.r.t. Abs

M-Atr	Abs. (AU) at different frequencies					
	3454	3266	3124	1622	1577	1550
Mn	1.01	1.28	0.67	0.58	0.64	0.89
Fe	0.41	0.37	0.18*	0.17	0.17	0.15
Co	0.99	0.67	0.27	0.28	0.38	0.42
Ni	0.61	0.67	0.22	0.30	0.39	0.56
Cu	0.48	1.07	0.52	0.54	0.66	1.01
Zn	1.76	1.16*	0.52*	0.24	0.28	0.30
Atr	0.12	0.31	0.15	0.19	0.62	0.33

* Not sharp peaks, but only shoulder observed

### FTIR analysis over a range of 1,800–1,350 cm^−1^

On the basis of Fig. 3 and Table 2, there were two peaks observed at 1,622 and 1,550 cm^−1^ due to bending frequencies of NH and stretching frequencies of C=N. The trends of vibrational modes of metal complexes were very similar to the order of stretching frequencies at NH. As mentioned above C=N is more basic in nature and it can coordinate easily with different metal ions. FTIR analysis at 1,550 cm^−1^ has shown that Cu-Atr has highest absorption and Fe-Atr has lowest absorption. The order of absorption of metal-Atr complexes at 1,550 cm^−1^ is; Cu > Mn > Ni > Co > Zn > Fe (Fig. 3). At 1,622 cm^−1^ the exact order of absorption is; Mn > Cu > Ni > Co > Zn > Fe. Similarly at 1,622 cm^−1^ the exact order of absorption is; Cu > Mn > Ni > Co > Zn > Fe.

### Structure prediction

From the analysis of all FTIR spectra, it is quite clear that there was involvement of crystalline (3,750–3,600 cm^−1^) and coordinated (3,500–3,430 and 1,625 cm^−1^) water molecules in the atrazine–metal complexes. To confirm the stoichiometry as well the presence of water and chloride ions, CHN analysis was also done (Table 3). It was observed that with Mn(II), Atr formed octahedral complex and coordinated through the C=N of ring. Here chlorine and water molecules are also acting as coordinated ions. Fe(II) ion has formed the distorted square planer complex in 1:1 where chlorine and water molecules were acting as coordinated ions. Co(II) ion has formed 1:1 distorted octahedral complex and coordinated through the C=N of ring and NH of side chain. Here chlorine and water molecules are also acting as coordinated ions. Ni(II) ion has formed the distorted square planer complex in 1:1 where chlorine and water molecules were acting as coordinated ions. Cu(II) ion has formed the distorted tetrahedral complex in 1:2 where chlorine and water molecules were acting as coordinated ions. Zn(II) ion has formed the distorted tetrahedral complex in 1:1 where only water molecules were acting as coordinated ions (Table 3).

**Table 3:** Most probable structures of metal complexes and CHN analysis

Metal ion	Molecular formula	Structure*	CHN analysis (%)					
			Cal.			Obs.		
			C	H	N	C	H	N
Mn	[Mn(Atr)_2_.(H_2_O)_2_(Cl)_2_].2H_2_O^a^	Octahedral	30.53	5.77	22.26	31.12	5.42	22.88
Fe	[Fe(Atr).(Cl)_2_].2H_2_O^b^	Square planar	25.39	4.79	18.50	25.56	4.65	18.59
Co	[Co(Atr).(H_2_O)_2_(Cl)_2_].2H_2_O^b^	Octahedral	23.01	5.31	16.77	23.14	5.36	16.68
Ni	[Ni(Atr).(H_2_O)(Cl)].2H_2_O^b^	Square planar	26.41	5.54	19.31	26.32	5.61	19.33
Cu	[Cu(Atr)_2_.(Cl)_2_].2H_2_O^a^	Tetrahedral	31.93	5.36	23.27	31.83	5.41	23.58
Zn	[Zn(Atr).(H_2_O)_2_].2H_2_O^b^	Tetrahedral	27.21	6.28	19.83	27.33	6.22	19.78

^a^Where Atr coordinated with metal ion(s) from CN side

^b^Where Atr coordinated with metal ion(s) from CN and NH side

* Most probable geometry

In previous experimental and theoretical studies of metal ions to atrazine, the complexation was affected by the water of hydration significantly, because, water molecule under high pH (5–8) values can act as good ligand, and in the presence of bulky molecules, it becomes more active to show coordination with metal ions (Martin et al. 1998; Medlycott et al. 2008; Meng and Carper 2000). The geometry, coordination and water of hydration of current study are very similar to previous experimental and theoretical studies. In previous experimental study, it observed that the metal ions were interacted to triazine (member of atrazine family) through the least bulky side chain nitrogen due to the steric effect of side chains (Martin et al. 1998; Medlycott et al. 2008; Meng and Carper 2000). In various theoretical studies it was mentioned that atrazine can form complex with transition and non-transition metal ion with variable number of atrazine and water molecules (Martin et al. 1998; Medlycott et al. 2008; Meng and Carper 2000). We have no particular explanation behind these structure arrangements, but it is quite understandable that if atrazine is forming monomeric complex with metal ion, the number of water molecules will increase. In liquid media (chloroform) metal effect of atrazine on the metal complexes of cadmium or copper or lead or zinc to 1-phenyl-3-methyl-4-p-tertbutylbenzoyl-5-hydroxypyrazole was studied (Martin et al. 1998; Medlycott et al. 2008; Meng and Carper 2000). It was observed that under liquid media (chloroform), atrazine can form complex with cadmium, copper and zinc with an order; Cd > Zn > Cu, this depends on the nature of the metal. In theoretical studies, it also has assumed that under liquid media (water) atrazine interacts with cadmium, copper and zinc by replacing one of the water molecules of the solvatation sphere. That mechanism might be explained by the “hard-soft theory” arguing that “soft” nitrogen atoms of atrazine have stronger interactions with “soft” cations (Martin et al. 1998; Medlycott et al. 2008; Meng and Carper 2000).

It is known that s-triazines (member of atrazine family) can be adsorbed on clay minerals as both protonated and neutral species. A comparison of the infrared spectra of atrazine adsorbed on montmorillonite with the spectrum of an acid solution of atrazine in water also revealed the presence of protonated species on the surface (Herwig et al. 2001; Bailey et al. 1968; Martin et al. 1998; Medlycott et al. 2008; Meng and Carper 2000). The sorption mechanism discussed was a cation exchange of the protonated species. Thus, in this case as well as for the ion–dipole or coordination-type interactions, it is to be assumed that the kind of surface cation plays a key role in the adsorption process. It was reported that at neutral pH ion–dipole interactions are in dominancy and coordination-type interactions under acidic conditions (Herwig et al. 2001; Bailey et al. 1968; Martin et al. 1998; Medlycott et al. 2008; Meng and Carper 2000).

### Molecular modeling

**Fig. 4:**
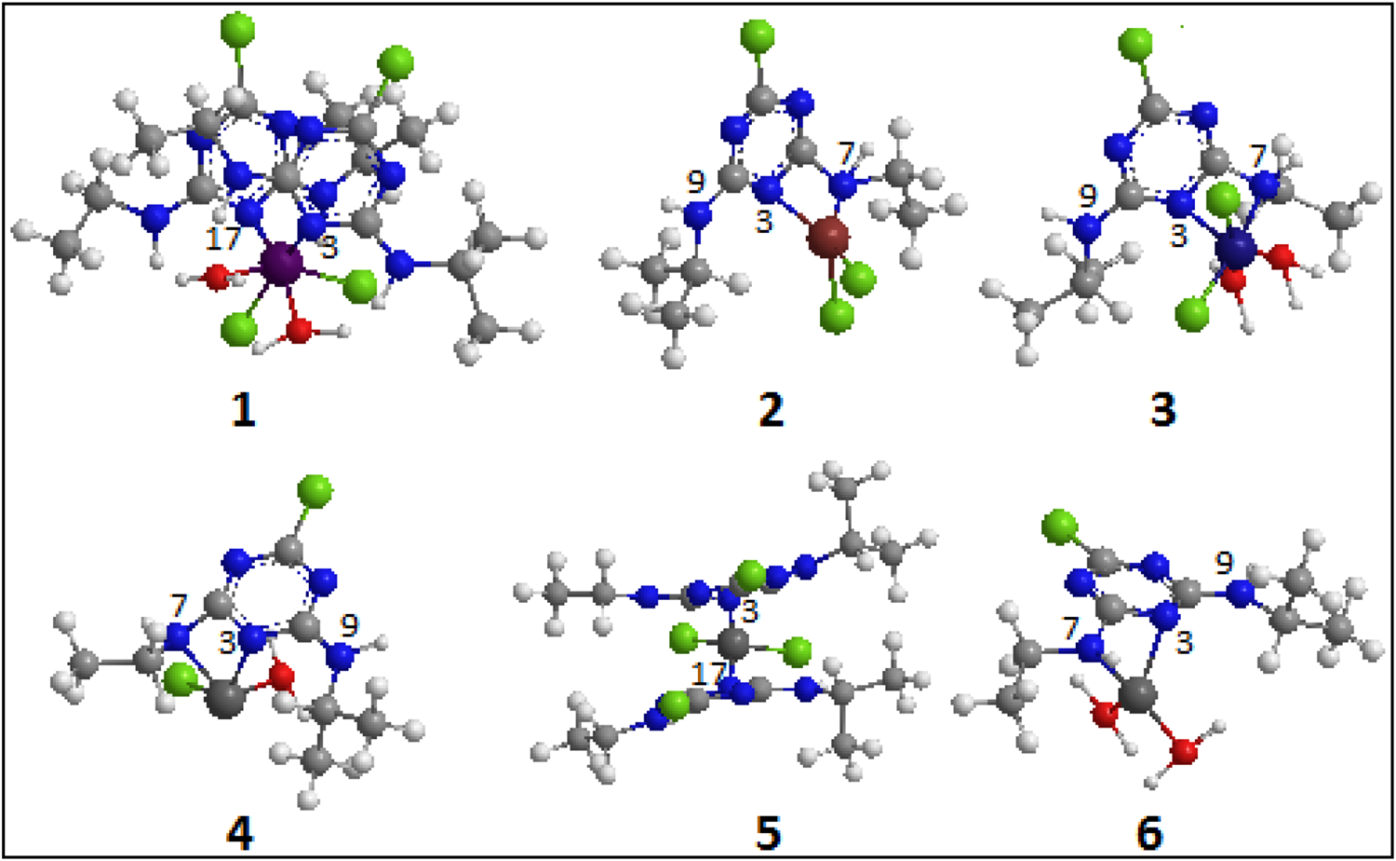
3D computational optimized structures of metal–Atrazine complexes

Since our trials to obtain a single crystals of the metal complexes were unsuccessful so far, and to gain a better understanding of geometrical structures of the investigated complexes, molecular modeling studies have been done by means of GAMESS (Cambridge Software ChemBio3D Ultra 14.0.) program package. Some selected bond lengths and angles are listed in Table 4; the optimized structures, with atom-labeling scheme, of complexes 1–6 are represented in Fig. 4. The complexes feature four and six coordinated metal centers with N of ring, N of side chain, Cl and H_2_O molecules. For complexes 1 and 5, the manganese and copper atoms are forming complexes with two Atr ligands through the N of ring. Whereas, for complexes 2, 3, 4 and 6, the metals are forming complexes with one Atr through the N of ring and N of less bulky chain including Cl and H_2_O as additional ligands to stabilize the metal complexes. The cis angles around the metal ions are variable, range from 74.17^o^ to 99.34^o^, respectively; the *trans* angles ranging from 130.26^o^ to 162.53^o^, respectively, indicating octahedral to square planar geometry with tetrahedral distortion (Al-Assy et al. 2013; Azahari et al. 2014; Bonora et al. 2013; de-Melo et al. 2014; Dong et al. 2014; Girichev et al. 2010; Kanagathara et al. 2013; Moghaddamand Foroushani, 2014; Yaremenko et al. 2008). For all complexes, the dihedral coordination around the metal center, involving O, N and Cl atoms is distorted. The degree of distortion from ideal tetrahedral geometry is given by the minimum and maximum coordination angles around metal ion. From Table 4, it is clear that the bond lengths are found to be within the normal ranges obtained from the crystal structure data of few nitrogen-based derivatives like triazines (Al-Assy et al. 2013; Azahari et al. 2014; Bonora et al. 2013; de-Melo et al., 2014; Dong et al. 2014; Girichev et al. 2010; Kanagathara et al. 2013; Moghaddamand Foroushani, 2014; Yaremenko et al. 2008). The obtained results are in a good agreement with the experimental results and hence strongly support them.

**Table 4:** 3D computational optimized bond lengths and bond angles of metal–atrazine complexes

Complex	Bond length/A^o^		Bond angle/degree (^o^)	
	Atoms	Bond length	Atoms	Bond angle
Mn(II)	C(2)-N(3)	1.283	N(3)-Mn(29)-O(32)	83.861
	C(2)-N(7)	1.276	N(3)-Mn(29)-O(33)	83.252
	N(17)-Mn(29)	1.883	N(3)-Mn(29)-Cl(30)	159.693
	N(3)-Mn(29)	1.882	N(3)-Mn(29)-Cl(31)	101.403
	Cl(30)-Mn(29)	2.180	N(3)-Mn(29)-N(17)	112.917
	Cl(31)-Mn(29)	2.174	N(17)-Mn(29)-Cl(30)	83.598
	O(32)-Mn(29)	1.870	N(17)-Mn(29)-O(33)	100.124
	O(33)-Mn(29)	1.866	N(17)-Mn(29)-Cl(31)	99.348
			N(17)-Mn(29)-O(32)	162.534
			Cl(30)-Mn(29)-O(33)	81.519
			Cl(30)-Mn(29)-Cl(31)	86.708
			Cl(30)-Mn(29)-O(32)	79.257
			O(33)-Mn(29)-Cl(31)	155.911
			O(33)-Mn(29)-O(32)	74.168
			Cl(31)-Mn(29)-O(32)	83.055
Fe(II)	C(2)-N(3)	1.260	C(2)-N(3)-C(4)	115.000
	C(2)-N(7)	1.266	C(2)-N(7)-C(8)	123.380
	N(7)-Fe(15)	1.409	N(7)-Fe(15)-Cl(16)	119.113
	N(3)- Fe(15)	1.846	Fe(15)-N(3)-C(4)	120.000
	Cl(16)-Fe(15)	2.160	Fe(15)-N(7)-N(3)	64.951
	Cl(17)-Fe(15)	2.160	Cl(16)-Fe(15)-Cl(17)	91.120
Co(II)	C(2)-N(3)	1.282	C(2)-N(3)-C(4)	119.160
	C(2)-N(7)	1.281	C(2)-N(7)-C(8)	106.970
	N(7)-Co(15)	1.878	N(7)-Co(15)-O(18)	97.376
	N(3)-Co(15)	1.882	Co(15)-N(3)-C(4)	125.317
	O(18)-Co(15)	1.163	Co(15)-N(7)-N(3)	65.179
	Cl(16)-Co(15)	2.180	Cl(16)-Co(15)-O(18)	166.722
Ni(II)	C(2)-N(3)	1.264	C(2)-N(3)-C(4)	120.186
	C(2)-N(7)	1.271	C(2)-N(7)-C(8)	115.207
	N(7)-Ni(15)	1.815	N(7)-Ni(15)-O(16)	108.722
	N(3)-Ni(15)	1.846	Ni(15)-N(3)-C(4)	122.919
	O(17)-Ni(15)	1.828	N(7)-Ni(15)-N(3)	63.301
	Cl(16)-Ni(15)	2.144	Cl(16)-Ni(15)-O(17)	76.316
Cu(II)	C(2)-N(3)	1.290	N(3)-Cu(29)-Cl(31)	104.712
	C(2)-N(7)	1.279	N(3)-Cu(29)-Cl(30)	112.655
	N(17)-Cu(29)	1.353	N(3)-Cu(29)-N(17)	124.741
	N(3)-Cu(29)	1.353	Cl(31)-Cu(29)-Cl(30)	92.278
	Cl(30)-Cu(29)	2.170	Cl(31)-Cu(29)-N(17)	114.992
	Cl(31)-Cu(29)	2.171	Cl(30)-Cu(29)-N(17)	102.823
Zn(II)	C(2)-N(3)	1.373	C(2)-N(3)-C(4)	122.097
	C(2)-N(7)	1.500	C(2)-N(7)-C(8)	136.829
	N(7)-Zn(15)	1.565	N(7)-Zn(15)-O(16)	136.826
	N(3)-Zn(15)	1.500	Zn(15)-N(3)-C(4)	144.251
	O(16)-Zn(15)	1.890	Zn(15)-N(7)-N(2)	86.348
	O(17)-Zn(15)	1.890	O(16)-Zn(15)-O(17)	118.507

## Conclusion

The IR spectroscopic studies of six new atrazine–metal complexes have shown that they have different structures consisting of one or two atrazine with one metal ion including variable water and chloride ions. Mn and Cu formed 1:2 complexes and coordination occurred through the nitrogen of ring. Remaining metal ions formed the 1:1 complexes by the involvement of nitrogen of ring as well as nitrogen of NH. The numbers of variable water and chlorine molecules were assigned using FTIR spectroscopy and CHN analysis. The apparent correlation between the M–N atrazine vibrations and the internal modes of atrazine offers a possible method of predicting metal–atrazine bond strength. The obtained results are supported by 3D molecular modeling of complexes.
